# Identification of Mushroom and Murine Tyrosinase Inhibitors from *Achillea biebersteinii* Afan. Extract

**DOI:** 10.3390/molecules26040964

**Published:** 2021-02-11

**Authors:** Marcelina Strzępek-Gomółka, Katarzyna Gaweł-Bęben, Apostolis Angelis, Beata Antosiewicz, Zuriyadda Sakipova, Kaldanay Kozhanova, Kazimierz Głowniak, Wirginia Kukula-Koch

**Affiliations:** 1 Department of Cosmetology, University of Information Technology and Management in Rzeszów, Sucharskiego 2, 35-225 Rzeszów, Poland; mstrzepek@wsiz.edu.pl (M.S.-G.); bantosiewicz@wsiz.edu.pl (B.A.); kglowniak@wsiz.edu.pl (K.G.); 2 Laboratory of Pharmacognosy and Natural Products Chemistry, School of Pharmacy, National and Kapodistrian University of Athens, Panepistimioupoli Zografou, 15771 Athens, Greece; aangjel@pharm.uoa.gr; 3 School of Pharmacy, Kazakh National Medical University Named after S.D. Asfendiyarov (KazNMU), 88 Tole Bi Street, Almaty 050012, Kazakhstan; sakipova.z@kaznmu.kz (Z.S.); kozhanova.k@kaznmu.kz (K.K.); 4 Chair and Department of Pharmacognosy, Medical University in Lublin, Chodźki 1, 20-093 Lublin, Poland; virginia.kukula@gmail.com

**Keywords:** *Achillea biebersteinii*, HPLC–MS analysis, tyrosinase inhibition, antioxidant properties, mushroom tyrosinase, murine tyrosinase, melanin release

## Abstract

Growing scientific evidence indicates that *Achillea biebersteinii* is a valuable source of active ingredients with potential cosmetic applications. However, the data on its composition and pharmacological properties are still insufficient. This study aims to optimize the extraction procedure of the plant material, evaluate its phytochemical composition, and compare anti-tyrosinase potential of *A. biebersteinii* extracts obtained by various methods. In order to identify compounds responsible for the tyrosinase inhibitory activity of *A. biebersteinii*, the most active anti-tyrosinase extract was fractionated by column chromatography. The fractions were examined for their skin lightening potential by mushroom and murine tyrosinase inhibitory assays and melanin release assay. HPLC-ESI-Q-TOF-MS/MS analysis of the total extract revealed the presence of several phenolic acids, flavonoids, flavonoid glucosides, and carboxylic acid. Among them, fraxetin-8-*O*-glucoside, quercetin-*O*-glucopyranose, schaftoside/isoschaftoside, gmelinin B, 1,3-dicaffeoylquinic acid (1,3-DCQA), and ferulic acid were found in the fractions with the highest skin lightening potential. Based on obtained qualitative and quantitative analysis of the fractions, it was assumed that the caffeoylquinic acid derivatives and dicaffeoylquinic acid derivatives are more likely responsible for mushroom tyrosinase inhibitory activity of *A. biebersteinii* extracts and fractions. Ferulic acid was proposed as the most active murine tyrosinase inhibitor, responsible also for the reduced melanin release from B16F10 murine melanoma cells.

## 1. Introduction

Nowadays, the ingredients used in cosmetic formulations are required to perform multiple functions and protect the skin from the harmful effects of the environmental factors. One of the most dangerous factors affecting the skin and stimulating the development of pathological conditions is ultraviolet radiation (UVR). Excessive exposure to UVR carries a significant health risk, including the development of skin cancer, as well as cause esthetic problems such as pigmentation disorders. Uneven skin pigmentation is caused by the dysregulation of the melanogenesis process by UVR, inflammation, hormonal imbalance, or several chemical compounds, including medicines [1]. The majority of the harmful effects of UVR are mediated by oxidative stress and subsequent damage of keratinocytes [2]. The most important mechanism of skin protection from the damage caused by UV radiation is melanin synthesis [3]. In response to oxidative stress, keratinocytes are known to secrete α-melanocyte-stimulating hormone (α-MSH) that induce melanogenesis by the surrounding melanocytes, thereby preventing the UVR-induced damage [4]. Due to the mechanism described above, the main targets of creams and ointments used for treating pigmentation disorders are reactive oxygen species (ROS) and tyrosinase (EC. 1.14.18.1), a copper-containing enzyme that catalyzes the first two rate-limiting steps of melanogenesis [5]. Thus, cosmetic ingredients that exhibit both antioxidant and skin-lightening properties are in high demand [6]. An interesting source of multifunctional ingredients for cosmetic formulations are plant extracts, rich in various active phytochemicals, especially polyphenolic compounds. The main benefits of plant extracts used in skin care products are antioxidant properties, antimicrobial activity, and inhibition of tyrosinase, leading to the reduction of hyperpigmentation disorders [7,8]. 

One of the particularly interesting source of antioxidant and antityrosinase cosmetic ingredients is *Achillea biebersteinii* Afan. (Asteraceae), a yellow flowering plant belonging to the same family (Astreaceae) as the common European medicinal plant *Achillea millefolium*. In traditional medicine, *A. biebersteinii* was used due to its wound-healing, antibacterial, and antifungal properties, and scientific evidence has also confirmed these in addition to its antioxidant, anti-inflammatory, and antinociceptive properties [9,10,11,12]. Methanolic extract from *A. biebersteinii* was recently identified as a potential source of antityrosinease compounds, with stronger mushroom tyrosinase inhibitory activity than *A. millefolium*, a common and valuable ingredient of natural cosmetics with skin-lightening and antioxidant properties [13]. Significant tyrosinase inhibitory properties were also recently described for hydroglycolic extracts from *A. biebersteinii*, inhibiting both monophenolase and diphenolase activity of this enzyme [14]. The most common phytoconstituents identified in *A. biebersteinii* extracts with tyrosinase inhibitory activity were chlorogenic acid, caffeic acid, rutin, quercetin, luteolin, apigenin [15], caffeoylquinic acid (CQA) isomers: 3-CQA, 4-CQA, 5-CQA, and a dicaffeoylquinic acid derivative: cynarin (1,3-DCQA) [14]. Some of these phytochemicals were already identified to inhibit melanin synthesis, but the compound responsible for tyrosinase inhibitory activity of *A. biebersteinii* extracts was not clearly identified to date. 

According to the regulatory frameworks governing the cosmetic industries in the United States and Europe, cosmetic products are required to be effective when used by consumers under normal, labeled, or foreseeable conditions. The claims for cosmetic products shall be supported by adequate and verifiable evidence, obtained using reliable and reproducible methodologies, with respect to the ethical conditions [16]. For that reason, the biological activity of novel active ingredients of cosmetics should be extensively studied. Whereas there are several experimental protocols allowing for assessing and confirming the antioxidant potential of synthetic or naturally-derived ingredients [17], searching for novel skin lightening compounds remains challenging. The method most commonly used to confirm skin lightening activity of plant extracts or compounds is an in vitro reaction when mushroom tyrosinase, isolated from *Agaricus bisporus*, is incubated with its substrate (L-tyrosine or L-DOPA), in the presence or absence of tested ingredients. The formation of colored reaction product (dopaquinone) is than measured by a spectrophotometer. Despite the simplicity, low costs, and high throughput of this method, it has several limitations, resulting from substantial differences between mushroom and mammalian tyrosinase [18,19]. Inhibitory activity of plant-derived extracts and compounds towards mushroom and mammalian tyrosinases may vary significantly [20]. For that reason, the skin lightening potential of novel active ingredients should be verified not only by the most commonly used mushroom tyrosinase inhibitory assays but also using mammalian sources of tyrosinase and cell culture experiments. In contrast to several studies confirming the inhibitory effect of *A. millefolium* on murine tyrosinase activity and melanin synthesis, skin lightening potential of *A. biebersteinii* extracts was studied only using mushroom tyrosinase inhibitory assay and has not been verified by other available experimental methods.

The aim of the present study was to evaluate the application of the extract of *A. biebersteinii* collected from the natural growth areas in the Almaty region, Kazakhstan as a potential antioxidant and tyrosinase-inhibitory ingredient for cosmetic formulations and to identify the constituents responsible for this action. The extraction conditions were optimized in order to obtain the fractions enriched in compounds with significant tyrosinase inhibitory properties. The skin lightening potential of the prepared extracts and fractions was evaluated using various experimental methods: mushroom tyrosinase inhibitory assay, murine tyrosinase inhibitory assay, and in vitro melanin release study.

## 2. Results and Discussion

### 2.1. Activity-Guided Optimization of A. biebersteinii Extraction Conditions

#### 2.1.1. The Influence of Extraction Conditions on Antioxidant Properties

**Figure 1:**
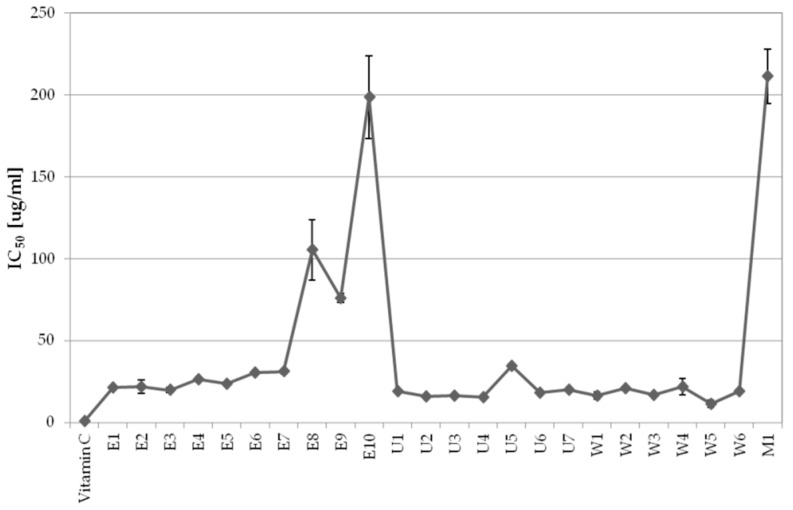
Antioxidant activity of *A. biebersteinii* extracts prepared using various extraction protocols, displayed as mean IC_50_ values ± SD obtained in DPPH scavenging assay; graph shows mean values ± SD, *n* = 3. E—ASE extracts, U—ultrasounds extracts, W—shaking extracts, M—maceration extract.

Dried aerial parts of *A. biebersteinii* were subjected to various extraction conditions in order to obtain the extract with the most significant cosmetic properties defined as strong antioxidant potential and tyrosinase inhibitory activity. The determination of the antiradical potential was conducted to find out how the extraction conditions affect the composition of extracts and as an introduction to further research on the whitening properties of the extracts. Antioxidant properties of the extracts were analyzed by DPPH scavenging assay, a reliable and reproducible method broadly used for evaluating the radical-scavenging activity of antioxidants. As shown in Figure 1, strong antioxidant properties were revealed by *A. biebersteinii* extracts obtained with the majority of techniques. For ultrasound assisted extraction the fractions (U1–U7) were characterized by their IC_50_ values: 15.6 ± 0.4 µg/mL for U4, 15.8 ± 0.7 µg/mL for U2 and 16.6 ± 0.4 µg/mL for U3; for shaking maceration fractions (W1–W6), the lowest IC_50_ values of W5 and W1 were: 11.5 ± 2.3 µg/mL and 16.5 ± 2.2 µg/mL, respectively; for Accelerated Solvent Extraction (ASE extracts, E1–E10) E3, E1, and E2 showed the IC_50_ of 19.8 ± 1.6 µg/mL, 21.4 ± 0.3 µg/mL, and 21.9 ± 4.1 µg/mL, respectively. It was clear that, in the case of ASE, the extraction time was significantly affecting the properties of the extract, which could be due to a prolonged heating process that could destroy components of the extract. As assumed maceration was the least effective extraction method with the weakest antioxidant activity (IC_50_ = 211.5 ± 16.5 µg/mL). Vitamin C used as a reference compound under the same conditions showed an IC_50_ value of 0.78 ± 0.05 µg/mL.

The antioxidant properties of *A. biebersteinii* alcoholic extracts were previously analyzed by Varasteh-Kojourian and co-workers using the DPPH scavenging assay, beta-carotene bleaching microplate assay, and TBARS test (using egg yolk homogenates as lipid-rich media) [21]. In this study, *A. biebersteinii* extracts showed stronger antioxidant activity than the extracts prepared from other *Achillea* species—*A. eriphora*. In this study, the IC_50_ value for *A. biebersteinii* extract obtained by the DPPH scavenging method was 276.0 ± 3.0 μg/mL. The difference between the IC_50_ value obtained by Varasteh-Kojourian and in this study may arise from the use of a different solvent (1:20 mixture of methanol and ethanol, w/v) [21]. In the other study, the antioxidant properties of *A. biebersteinii* extracts prepared from flowers, leaves, and roots were compared. The IC_50_ values in DPPH scavenging assay of 670.0 ± 44.0, 377.0 ± 11.0, and 773 ± 53.0 μg/mL, respectively, indicated that antioxidant compounds are the most abundant in *A. biebersteinii* leaves [15]. Lower IC_50_ values than in the presented work may be explained by the different collection sites of the plant material. Şabanoğlu and co-workers collected *A. biebersteinii* in Yahyali-Kayseri in Turkey, whereas the plant for this study was collected in the Almaty region in Kazakhstan, known for its severe climatic conditions. Strong influence of the collection site on the phytochemical content and chosen biological properties of plant extracts were previously shown for other species, including blackberries, grapes, and barberries [22,23,24]. Based on this evidence, it could be concluded that the plant material collected from locations with less favorable water and temperature conditions are possibly more diverse in their chemical composition and therefore more promising from a pharmacological point of view.

#### 2.1.2. The Influence of Extraction Conditions on Tyrosinase Inhibitory Properties

**Figure 2:**
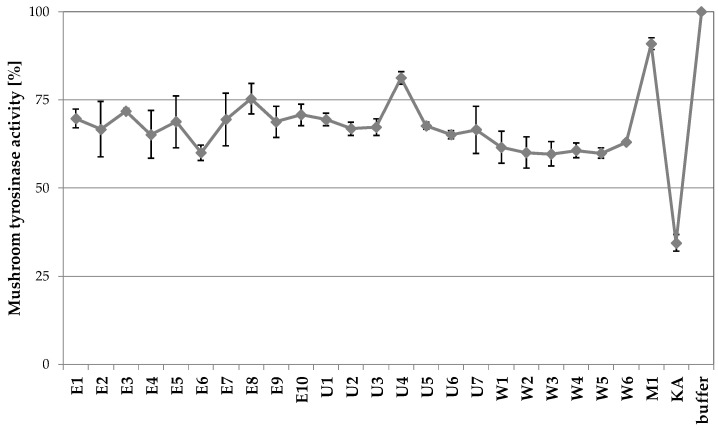
Mushroom tyrosinase inhibitory properties of *A. biebersteinii* extracts (100 µg/mL) prepared using various extraction protocols; 100 µg/mL kojic acid (KA) was used as a positive control; graph shows mean values ± SD, *n* = 3. E—ASE extracts, U—ultrasounds extracts, W—shaking extracts, M—maceration extract.

Among *Achillea* species, *A. millefolium* is the most known source of skin lightening compounds [13,25]. Tyrosinase inhibitory activity was also detected in extracts from aerial parts of *A. monocephala, A. coarctata* [26], *A. phrygia* [27], *A. alpina* [28], and *A. teretifolia* [13]. As previously summarized, *A. biebersteinii* extracts were recently reported as sources of tyrosinase inhibitors [13,14]. The results presented in Figure 2 confirmed previous findings, showing that most of the *A. biebersteinii* extracts decrease the activity of mushroom tyrosinase by 24–40%. The weakest mushroom tyrosinase inhibitory activity was reported for the extract prepared by maceration (9% inhibition), whereas the highest inhibitory activity was found for the E6 extract, prepared by ASE (ca. 40% inhibition).

In order to verify and complete the previously published findings on novel tyrosinase inhibitors present in *A. biebersteinii,* extract E6, prepared by ASE, was chosen for further fractionation and analysis. The aim of the further study was to identify the active compound responsible for mushroom tyrosinase inhibition as previous research identified only major components of the extracts with high anti-tyrosinase activity [13,14]. Additional studies, involving mammalian tyrosinase, were also performed in order to confirm the skin lightening potential of *A. biebersteinii* compounds.

### 2.2. The Identification of Active Components in EtOH Extracts from Achillea biebersteinii by HPLC and HPLC–ESI-Q-TOF-MS

**Figure 3:**
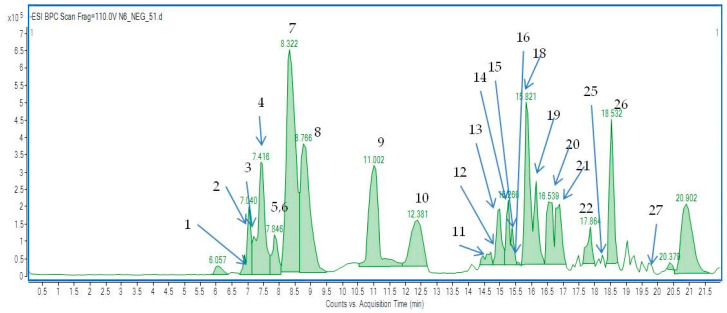
The TIC chromatogram recorded in the negative ionization modes for the *Achillea biebersteinii* E6 extract.

The secondary metabolites present in *A. biebersteinii* extract E6 were identified by HPLC–ESI-Q-TOF-MS. The identification was performed based on the scientific literature, commonly available fragmentation bases (METLIN), comparison of retention times, and data from the high-resolution mass spectrometer used in the study. Table 1 presents the constituents tentatively identified in the *A. biebersteinii* extracts. Even if some compounds were identified in both ionization modes, the negative ionization was found to detect a larger majority of metabolites in the tested extracts (see Figure 3 and for the positive ionization mode—Figure S1 in the Supplementary File). The operation parameters applied for HPLC–ESI-Q-TOF-MS instrument enabled the ionization of the secondary metabolites from the *A. biebersteinii* extracts and led to the formation of sharp peaks and a stable baseline for all injections (Figure 3).

Based on the high resolution MS data, 27 secondary metabolites belonging to different groups such as flavonoids, tannins, and phenolic acids and their ester derivatives were identified. The following major components of the E6 were identified: 3-caffeoylquinic acid (3-CQA), 4-caffeoylquinic acid (4-CQA), 5-caffeoylquinic acid (5-CQA), achillin, apigenin, axillarin, caffeic acid, caffeoylglucoside isomers, citric acid and isocitric acid, coumaroylquinic acid isomers, 1,3-dicaffeoylquinic acid (1,3-DCQA), ellagic acid, ferulic acid, fraxetin-8-*O*-glucoside, gluconic acid, glutaric acid, gmelinin B, isovitexin, jaceidin, kaempferol, malic acid, protocatechuic acid glucoside, quercetin-*O*-glucopyranose, quinic acid, schaftoside or isoschaftoside, shikimic acid, and succinic acid. 

Phytochemical composition of *A. biebersteinii, A. setacea*, and *A. wilhelmsii* extracts was previously performed by Şabanoğlu et al. using RP-HPLC-DAD. This analysis revealed the presence of rutin, luteolin, apigenin, and quercetin in these plants. In addition, several phenolic compounds including chlorogenic acid and caffeic acid were identified in *Achillea* extracts [15]. Hyun Joo Lee et al. explored the phytochemical content of *A. alpina* and identified the following components: jaceidin, axillarin, 5,7,4-trihydroxy-3,6-dimethoxyflavonone, isoshaftoside, shaftoside, neoshaftoside, isovitexin, quercetin, chlorogenic acid, chlorogenic acid methyl ester, 5-*O*-coumaroylquinic acid, 5-*O*-coumaroylquinic acid methyl ester, 3,5-DCQA acid, methyl 3,5-DCQA acid, penduletin, chrysosplenol B, 3-*O*-vicianoside, and achillin [28]. These compounds were also intensified by Ali S.I. et al. in *A. millefolium* extracts along with guaianolides (8-hydroxyachillin, austricin, desacetylmatricarin, digydroparthenolide, dihydroreynosin, isopaulitin, millefin, paulitin, *α*-peroxyachifolid, psilostachyin, sintenin, rupicolin A and B) and phytosterols (campesterol, cholesterol, stigmasterol and β-sitosterol) [34]. Zengin and co-authors (2017) identified caffeic acid, 3- and 4-caffeoylquinic acid, 1,3-dicaffeoylquinic acid, 3,5-dicaffeoylquinic acid, 4,5-dicaffeoylquinic acid and 3,4,5-tricaffeoylquinic acid in *Achillea biebersteinii*. The other chemical compounds that are present in *A. biebersteinii* are: 1- and 4-feruloquinic acid and protocatechuic acid [13]. Gaweł-Bęben and co-authors identified 1,3-dicaffeoylquinic acid, 3-, 4-, 5-caffeoylqunic acid, caffeic acid, kaempferol, jaceidin, axillarin, coumaroyl-quinic acid isomers, quinic acid, and 3,8-dimethylherbacetin [14]. 

### 2.3. The Fractionation of E6 Extract

The E6 extract that was found to be the most active in the tyrosinase inhibitory assay was fractionated by column chromatography as described in the ‘Materials and Methods’ section. All fractions were directed to the composition studies and tyrosinase inhibition test with mushroom tyrosinase. The identification of the components from the three most active fractions was evaluated by HPLC-MS.

#### 2.3.1. Tyrosinase Inhibitory Activity of *A. biebersteinii* Fractions

**Figure 4:**
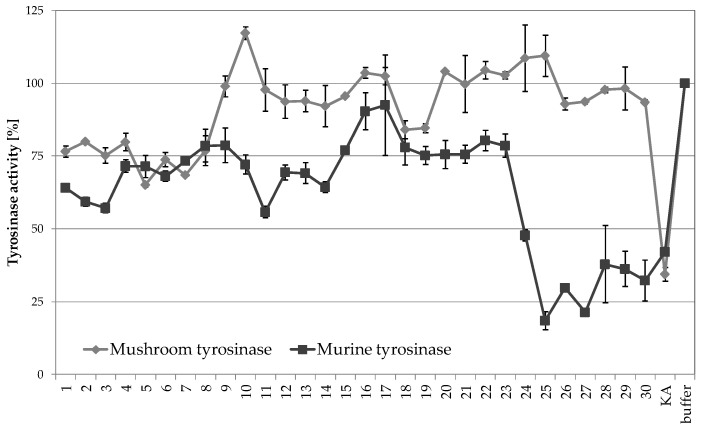
Mushroom and murine tyrosinase inhibitory properties of *A. biebersteinii* fractions (100 µg/mL) prepared using various extraction protocols; 100 µg/mL kojic acid (KA) was used as a positive control; graph shows mean values ± SD, *n* = 3.

The fractions of the E6 extract were analyzed for their inhibitory properties towards mushroom tyrosinase. The most significant mushroom tyrosinase inhibitory activity was found in fractions 5, 6, and 7, displaying 34.9, 24.3, and 31.5% inhibitory activity, respectively (Figure 4). The fractions were than analyzed for their inhibitory properties towards murine tyrosinase, obtained from B16F10 murine melanoma cell lysate. The structural features of mushroom and mammalian tyrosinases are significantly different (mushroom tyrosinase is a monomeric enzyme present in the cytoplasm, whereas mammalian tyrosinases are tetrameric, transmembrane proteins located in melanosomes), resulting in major differences in their activity [19]. Significant differences in the inhibitory activity towards mushroom and murine tyrosinases were previously described for other naturally derived compounds such as p-coumaric acid [35] and aloesin [36]. In the performed analysis, murine tyrosinase was significantly inhibited by most of the E6 extract fractions (with exception of fractions 16 and 17). The most significant murine tyrosinase inhibitory activity of about 80% was detected in fractions 25 and 27 at 100 µg/mL concentration (Figure 4). The inhibitory properties of fractions 25 and 27 were higher than of a control tyrosinase inhibitor—kojic acid, at the same concentration. Interestingly, these fractions showed no significant inhibitory activity towards mushroom tyrosinase.

#### 2.3.2. The Influence of *A. biebersteinii* Fractions of Melanin Content and Viability of B16F10 Cells

**Figure 5:**
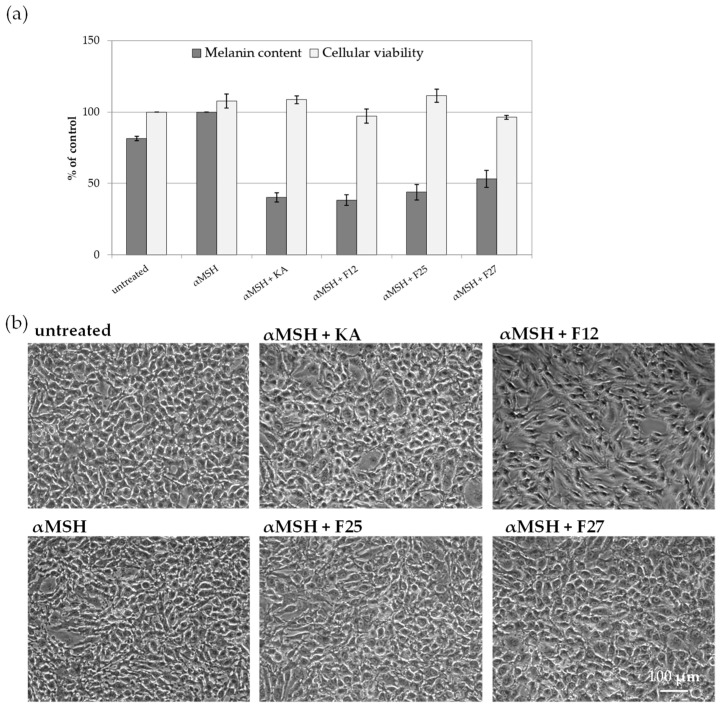
The influence of *A. biebersteinii* fractions on melanin content and viability of B16F10 murine melanoma cells. (**a**) The relative amount of melanin release into the conditioned medium was measured following 72 h of culture ± αMSH and 100 µg/mL F12, F25 or F27 *A. biebersteinii* fractions. Cellular viability was assessed by the Neutral Red Uptake Test. The graph shows mean ± SD, *n* = 2; (**b**) morphology of B16F10 cells following 72 h of culture ± αMSH and F12, F25, or F27, 10× magnification, pictures are representative for two independent experiments.

The efficacy of obtained *A. biebersteinii* fractions as inhibitors of melanin synthesis was further investigated in vitro, using B16F10 murine melanoma cells. This cell line is known to synthesize and release significant amounts of melanin and therefore is a practical model to study melanogenesis inhibitors. For this study, three *A. biebersteinii* fractions were chosen: 25 and 27, with the strongest murine tyrosinase inhibitory activity and 12, with moderate inhibitory potential. As shown in Figure 5, all three analyzed fractions decrease the release of melanin into the conditioned medium of B16F10 cell stimulated with α-MSH, a physiological regulatory hormone activating melanogenesis. At the same time, the viability of the cells was not affected by these treatments. However, microscopic analysis of cellular morphology revealed that fraction 12 visibly changed cellular morphology. This observation, combined with the moderate activity of fraction 12 in murine tyrosinase inhibitory assay, may suggest that the reduced melanin release from cells treated with this fraction results not only from tyrosinase inhibition, but also impaired cellular function.

#### 2.3.3. HPLC-ESI-Q-TOF-MS/MS—Based Identification of the Antityrosinase Constituents of the Fractions

**Figure 6:**
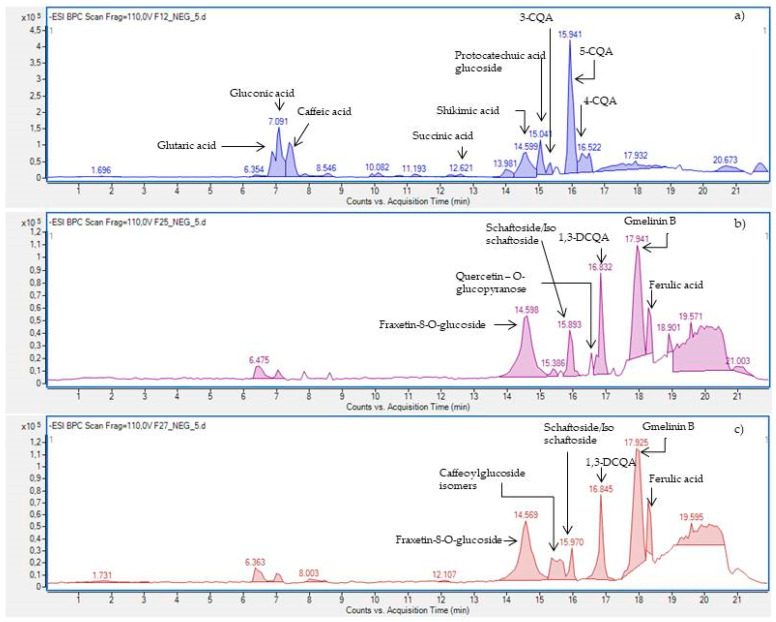
The TIC chromatogram recorded in the negative ionization modes for the *Achillea biebersteinii* fractions: 12 (**a**), 25 (**b**), and 27 (**c**).

Based on the results of anti-tyrosinase assays, the three most active fractions were selected for qualitative analysis by HPLC-ESI-Q-TOF-MS/MS in order to identify compounds responsible for the observed biological effect. The composition of fractions 12, 25, and 27, visualized in Figure 6, shows the presence of caffeic acid derivatives, simple organic acids, and some flavonoid derivatives that could be responsible for the whitening effect of the *A. biebersteinii* extract (for the identification details, see Table 1 and Table S1 from the Supplementary Material).

Caffeic acid derivatives that were identified in the most active fractions F25 and F27 were previously suggested by Zengin and co-workers as the most important compounds responsible for the mushroom tyrosinase inhibitory activity of *A. biebersteinii* extracts [13]. Careful scientific literature screening allowed us to find more data on potential skin lightening activity of caffeic acid derivatives. The study of Kazuya and collaborators proved that 3,4-DCQA, 3,5-DCQA, and 4,5-DCQA at the concentration of 90 µM strongly inhibited the formation of dopachrome from L-tyrosine (45–50%) and from L-DOPA (51–59%) in the mushroom tyrosinase inhibitory assay [37]. In another study using B16F10 cells stimulated with α-MSH, the two derivatives of caffeic acid: 1,5-DCQA and 4,5-DCQA decreased melanin release by 61% and 84%, respectively. Moreover, 4,5-DCQA downregulated the expression of microphthalmia-associated transcription factor (MITF) responsible for the transcription of tyrosinase gene as well as tyrosinase-related protein 1 (TRP1), involved in the regulation of the melanogenesis pathway. The effect of 4,5-DCQA was mediated by decreased generation of cAMP and inhibition of cAMP response element-binding protein (CREB) phosphorylation [38]. The information summarized above strongly indicates that the skin lightening potential of *A. biebersteinii* extracts and fractions, rich in various caffeic acid derivatives, may regulate the melanin synthesis not only by simple tyrosinase inhibition, but also at the gene expression level.

In the light of our findings and the data previously published by Zengin and collaborators [13] who proved anti-tyrosinase potential of *A. biebersteinii* extracts, the authors of this manuscript found it important to quantify the content of the caffeic acid derivatives in the investigated fractions. The compounds found in *A. biebersteinii* extracts that may contribute to the detected melanogenesis inhibitory activity to the highest extent include 3-CQA, 5-CQA, 4-CQA, and 1,3-DCQA. Their content in the fractions was calculated based on a direct comparison with relevant reference compounds’ injections at the same analytical conditions. Fraction F12 contained 0.205 ± 0.008% of 3-CQA, 2.187 ± 0.12% of 4-CQA, and 0.529 ± 0.018% of 5-CQA; fraction F25 4.363 ± 0.12% of 1,3-DCQA and fraction F27: 4.038 ± 0.22% of 1,3-DCQA.

Based on these calculations, the F25 fraction was found to contain a higher quantity of 1,3-DCQA from the fraction F27. In addition, F25 contained a marked concentration of the caffeoylglucoside isomer of unknown identity that could additionally influence the total whitening activity of the fraction.

Even if the DCQA derivatives were proved to exhibit the strongest tyrosinase inhibitory properties in the previously published articles, interestingly, our study showed that fraction 12 was also found to be active even if it did not contain the DCQA derivatives, but simple organic and phenolic acids. Based on these observations, it could be concluded that these low molecular mass polar constituents are also important inhibitors of melanin production.

These assumptions were confirmed also by other researchers. Ferulic acid (0.31 mM) and caffeic acid (0.15 mM) were proved to be efficient in the pigment whitening process by Maruyama and collaborators. These compounds decreased the melanin levels in B16F10 melanoma cells by 27.4% and 24.4%, respectively. Furthermore, in a murine tyrosinase inhibitory assay, ferulic acid inhibited the conversion of tyrosine to L-DOPA by 98% (51.5 µM) and caffeic acid suppressed the same conversion by 15%. Probably, the mechanisms of decreased activity of tyrosinase are different for ferulic acid and caffeic acid. Ferulic acid showed direct binding to tyrosinase and thus inhibited melanin production. On the other hand, caffeic acid did not show direct binding to tyrosinase [39]. In other studies, ferulic acid decreased melanin content in B16 F10 melanoma cells in a concentration dependent manner by 13.9%, 25.5%, and 43.6% (at 5, 10, and 20 µg/mL, respectively). Ferulic acid was also shown to downregulate the expression of tyrosinase and MITF proteins in murine cells (26.7% of inhibition at the concentration of 20 µg/mL) [40]. In our studies, the level of ferulic acid in the fractions F25 and F27 was calculated as 0.398 ± 0.01% and 0.473 ± 0.013%, respectively. Other compounds identified in F25 and F27 fractions with skin lightening potential described in the literature include shikimic acid and schaftoside. The zebrafish whitening drug screen test proved that shikimic acid decreased the melanin content of zebrafish embryos. Shikimic acid at the concentration of 500 µM reduced the pigmentation area in zebrafish embryos of 78%. Furthermore, shikimic acid exhibited tyrosinase inhibitory effect in B16 cells. In the experiment on the cell lines, shikimic acid at the concentration of 500 µM decreased the activity of tyrosinase of 85% after 72 h [41]. Schaftoside, also found in fractions 25 and 27, showed anti-melanogenic effects in α-MSH stimulated B16F1 cells. Interestingly, Kim et al. proved that the treatment with schaftoside noticeably decreased the expression level of tyrosinase and TRP1 in B16F1 cells [42].

**Figure 7:**
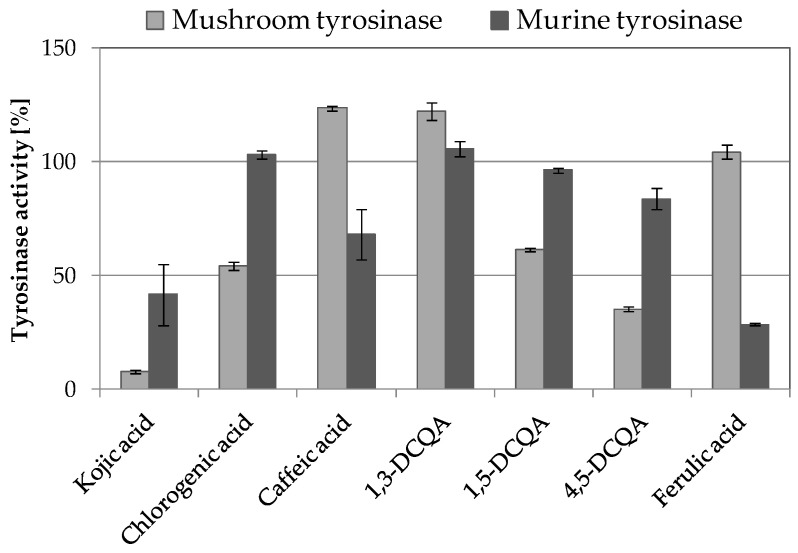
Mushroom and murine tyrosinase inhibitory activity of 100 µg/mL pure major compounds identified in F12, F25, and F27 *A. biebersteinii* fractions; 100 µg/mL kojic acid (KA) was used as a positive control; graph shows mean values ± SD, *n* = 3.

In order to make a final conclusion about the compounds responsible for detected tyrosinase inhibition and decreased melanin production in B16F10 cells, the mushroom and murine tyrosinase inhibitory activity assays of major compounds identified in fractions F12, F25, and F27 were performed (Figure 7). 1,5-DCQA and 4,5-DCQA were found to be the most potent mushroom tyrosinase inhibitors among analyzed compounds, decreasing the activity of mushroom tyrosinase by 38.4% and 64.9%, respectively. Interestingly, these compounds showed only weak inhibitory activity against murine tyrosinase − 3.7% and 13.3%. 1,3-DCQA showed no inhibitory activity towards mushroom and murine tyrosinase at the tested concentration. The strongest murine inhibitory activity was reported for ferulic acid, reducing the activity of murine tyrosinase by 71.4%. At the same time, ferulic acid did not influence the activity of mushroom tyrosinase.

Considering the above information and analyzing the compositional data with this study, we could conclude that di-caffeoylquinic acids are potent inhibitors of mushroom tyrosinase and thus confirm the findings previously published by Zengin and co-workers [13]. However, di-caffeoylquinic acids did not significantly influence the activity of murine tyrosinase and their melanogenesis inhibitory activities reported previously by Kazuya [38] and Ha [39] resulted in being more likely from other mechanisms than direct tyrosinase inhibition, including downregulation of TRP1 and MITF gene expression.

Our studies using pure compounds strongly indicate that ferulic acid is the most active murine tyrosianse inhibitor present in *A. biebersteinii* extracts. This compound was present in the most active F25 and F27 fractions but not in fraction F12. The decreased melanin release from F12-treated B16F10 cells might result from the impaired cellular function (suggested by the changed cellular morphology) or the influence of other compounds found in this fraction on various levels of the complex melanogenesis process.

The presented results also prove that the selected experimental model plays a key role in the research on novel skin lightening compounds due to the significant differences between mushroom and murine tyrosinase activities. 

## 3. Materials and Methods 

### 3.1. Chemicals

96% ethyl alcohol and methanol of reagent grade were purchased from Avantor Performance Materials (Gliwice, Poland). Mushroom tyrosinase from *Agaricus bisporus*, DPPH (2,2-diphenyl-1-picrylhydrazyl), L-DOPA (L-3,4-dihydroxyphenylalanine), NaH_2_PO_4_ · 2H_2_O, Na_2_HPO_4_ · 2H_2_O, dimethyl sulfoxide (DMSO), kojic acid (KA) and reference compounds for quantitative analysis purity exceeding 95% (caffeic acid, chlorogenic acid, 3-caffeoylquinic acid, 5-caffeoylquinic acid, 4-caffeoylquinic acid, ferulic acid, 1,3-DCQA, 1,5-DCQA, 4,5-DCQA and quercetin, Dulbecco’s Modified Eagles Medium (DMEM), Dulbecco’s Phosphate Buffer Saline (DPBS), neutral red, α-melanocyte stimulating hormone (α-MSH), Triton X-100 were obtained from Sigma Aldrich (St. Louis, MO, USA). Fetal Bovine Serum (FBS) was obtained from PAN Biotech. DC Protein Assay was obtained from Bio-Rad (Warszawa, Poland). B16F10 murine melanoma cell line (ATCC CRL-6475) was purchased from LGC Standards (Łomianki, Poland).

### 3.2. Plant Material

Plant material—the aerial flowering parts of *Achillea biebersteinii*—was collected in Kazakhstan, in the Pavlodar region, in the outskirts of Bayanaul village. Professor Zuriyadda Sakipova recognized the species of *A. biebersteinii* and collected the flowering overground parts of the plant in May 2018, drying them in the shade at the temperature not higher than 30 °C.

### 3.3. The Optimization of Extraction

Four extraction techniques were implemented to evaluate the optimal conditions suitable for the extraction of *A. biebersteinii* metabolites from the plant matrix. All of them were planned to sustain the 1:10 solid:liquid ratio and to be comparable between one another. The solvents used for extraction included distilled H2O, ethanol, 75% (*v*/*v*) ethanol, 50% (*v*/*v*) ethanol, and 25% (*v*/*v*) ethanol. After the extraction, all extracts were filled to the volume of 25 mL with the extracting solvent, filtered through a nylon syringe filter (pore diameter of 0.45 µm, Merck Millipore, Darmstadt, Germany) and used for the qualitative and quantitative analyses on the HPLC-ESI-Q-TOF-MS platform. The extracts were prepared in triplicate. The remaining extracts were evaporated to dryness using a rotary evaporator at 40 °C and used for the biological assays. The extraction conditions are summarized in Table 2.

#### 3.3.1. Accelerated Solvent Extraction (ASE)

An ASE 100 apparatus (Dionex, Sunnyvale, CA, USA) with 10 mL stainless steel vessels was used for the pressurized liquid extractions. In addition, 1 g of freshly powdered *A. biebersteinii* was placed in an extraction cell each time. Following the addition of solvents, the cell was pressurized, heated, and extracted statistically under the following conditions: static time: 5 min for extracts E1–E7, 2 min for extract E8, 7 min for extract E9, 10 min for extract E10, number of cycles: 3, temperature: 60 °C for E1, 80 °C for E2, 100 °C for E3, 120 °C for E4, 140 °C for E5, 160 °C for E6, 180 °C for E7, 80 °C for E8, E9, and E10, flush volume for extracts E1 60%, 30% for extracts E2–E10, flushing time: 10 s.

#### 3.3.2. Maceration

One gram of powdered *A. biebersteinii* was macerated in 75% (*v*/*v*) ethanol in the glass vials for 5 h. The plant material was gently mixed with a glass spoon every half an hour. The derived extract was filtered through a hard filter paper. 

#### 3.3.3. Ultrasonic Extraction

0.5 g of *A. biebersteinii* powder was extracted in the glass vials using an ultrasonic bath (Sonic-3, Polsonic, Warsaw, Poland). Following the addition of appropriate solvent (Table 2), aliquots were extracted in changing conditions. After the extraction, the extracts were filtered through a hard filter paper and evaporated to dryness using a rotary evaporator at 40 °C.

#### 3.3.4. Shaking Extraction

Samples of 0.5 g of *A. biebersteinii* powder were vigorously shaken (300 rpm) in the glass vials for the following periods of time: 5 min, 10 min, 15 min, 20 min, 25 min, and 30 min (Table 2) and filtered using a hard filter paper.

### 3.4. HPLC-ESI-Q-TOF-MS/MS Analysis of the Extracts

A qualitative analysis of the extracts and the obtained fractions together with quantitative studies on the caffeic acid derivatives present in the tested samples was performed on an Agilent Technologies liquid chromatograph coupled with an electrospray–quadrupole–time of flight-mass spectrometer (HPLC-ESI-Q-TOF-MS/MS). The platform was composed of an HPLC chromatograph (Agilent 1200 Series) with a binary pump, a degasser, a photodiode array detector, a column thermostate, an autosampler, and of a Q-TOF MS (6500 Series) (Aligent Technologies, Santa Clara, CA, USA) spectrometer with an ESI spray ionization method. Then, the extracts were filtered through a syringe nylon membrane filter and were directed to the fast gradient-based separation method on the Zorbax RP18 HPLC column (150 × 2.1 mm, d = 3.5 μm, Agilent Technologies, Santa Clara, CA, USA). The mobile phase consisted of 0.1% formic acid (Solvent A) and acetonitrile with 0.1% formic acid (Solvent B). The following conditions were set: 5.0% A and 95.0% B for the first 10 mins, 99.0% A and 1.0% B reached until the 12th minute and later kept for the following 6 min. The flow rate was set to 0.2 mL/min, the injection volume was 5.0 μL, and the total analysis time was 22 min. All UV spectra were collected within a wavelength range from 190 nm to 500 nm, namely: 201, 254, 280, 290, 320, 365 nm. The ESI source was operated at a drying gas flow of 12 mL/min, nebulizer of 35 psi, fragmentor voltage of 110 V and skimmer voltage of 65 V. Two collision energies were applied (10 and 20 V) to provide the MS/MS spectra. The temperature of capillary and vaporizer was 325 °C. The sheath gas flow was set at 12 L/min and the temperature of 350 °C. All spectra were acquired in both positive and negative ionization modes in the mass range of 50–1200 *m*/*z*. The MassHunter version B.08.00 software (Aligent Technologies, CA, USA) was used for the administration of all MS spectra of the studied extracts. The suggested molecular formulas were confronted with the constituents described in the scientific literature to achieve the identification of the major components of the examined extracts. For the quantitative assay, the calibration curves of the relevant standards were prepared from 5 independent concentrations of each standard compound, and they were injected at the volume of 10 µL each, to draw the 5-point calibration curves. The quantitation of caffeic acid derivatives and ferulic acid in the fractions was performed based on triple injection of each analyzed fraction and an additional fourth one performed on a consecutive day.

### 3.5. Fractionation of the Extract 

The E6 extract that was found to be the most active in the tyrosinase inhibitory assay was fractionated by column chromatography using LH-20 Sephadex adsorbent (Sigma Aldrich, St. Louis, MO, USA). The column chromatography provided 30 fractions 10 mL each. The elution with 50% methanol provided fractions 1–17. Methanol was used to elute the fractions 18–30 from the column. All fractions were filtered through a nylon syringe filter (0.45 µm, Merck Millipore, Darmstadt, Germany) and evaporated to dryness using a rotary evaporator at 45 °C prior to the composition studies and a tyrosinase inhibition test with mushroom tyrosinase. The composition of the three most active fractions was evaluated by HPLC-MS. 

### 3.6. DPPH Scavenging Assay

All extracts prepared by ASE, maceration, ultrasounds, and shaking were assayed for their ability to quench free radicals in a 1,1-diphenyl-2- picrylhydrazyl (DPPH) test. The procedure described by Matejic et al. was applied in the studies after slight modifications [43]. In addition, 100 μL of appropriately diluted extract (1 mg/mL, 0.5 mg/mL, 0.25 mg/mL, 0.125 mg/mL, 0.0625 μg/mL, 0.03125 mg/mL, 0.01563 mg/mL, 0.00781 mg/mL, 0.00391 mg/mL, 0.00195 mg/mL, 0.00098 mg/mL, and 0.00049 mg/mL) or pure solvent for the control sample was mixed with 100 μL DPPH˙ (25 mM in 99.9% methanol). After 20 min of incubation at room temperature in darkness, the absorbance of the samples was measured at λ = 540 nm using a microplate reader (FilterMax F5 Molecular Devices, San Jose, CA, USA). The obtained values of measurements were corrected by the absorbance value of the extract without DPPH. Furthermore, 100 μL water with DMSO (1:1 *v*/*v*) mixed with 100 μL DPPH˙ was used as a control sample. The analysis was conducted in 3 independent repetitions, using vitamin C as a reference compound. The percentage of DPPH˙ scavenging power was calculated for each sample based on the following equation:

% of DPPH˙ scavenging = [1 − (AS/AC)] × 100(1)

where AS is the corrected absorbance of the sample and AC is the corrected absorbance of the control sample.

### 3.7. Mushroom Tyrosinase Inhibitory Assay

Inhibition of mushroom tyrosinase by *A. biebersteinii* extracts was performed using the method described previously by Uchida and co-investigators [44]. Briefly, 120 μL phosphate buffer (100 mM, pH = 6.8) was mixed with 20 μL sample solution or pure compounds (1 mg/mL) and 20 μL of mushroom tyrosinase (500 U/mL) and pre-incubated at room temperature for 10 min. Following the addition of 40 μL L-DOPA (4 mM), the samples were incubated for another 20 min at RT. The dopachrome formation was measured spectrophotometrically at λ = 450 nm using FilterMax F5 microplate reader (FilterMax F5 Molecular Devices, San Jose, CA, USA). The obtained values were corrected by the absorbance value of the extract without mushroom tyrosinase and L-DOPA. At the same time, DMSO, solubilized in adequate concentrations in phosphate buffer, was added and used as a positive control relevant to the 100% tyrosinase activity. Each sample was analyzed in 3 independent repetitions.

### 3.8. Murine Tyrosinase Inhibitory Assay

Inhibition of murine tyrosinase by *A. biebersteinii* extracts was assessed using B16F10 murine melanoma cell lysate as described by Uchida et al. with slight modifications [44]. B16F10 were maintained in DMEM high glucose supplemented with 10% FBS in humidified atmosphere at 37 °C and 5% CO_2_. In addition, 8 × 10^6^ cells were lysed in 5 mL of 100 mM phosphate buffer pH 6.8 with 1% Triton X-100 in a sonicator ice cold water bath for 1 h. Following centrifugation (10 min, 13,000 rpm), the supernatant was collected as murine tyrosinase solution and the protein concentration was measured using a DC Protein Assay. To establish the inhibitory activity of *A. biebersteinii* extracts, the volume of cell lysate containing 20 µg protein was mixed with 20 µL sample or pure compounds, 40 µL 4 mM L-DOPA, and 100 mM phosphate buffer pH 6.8 (up to 200 µL). The absorbance at λ = 450 nm was measured following 4 h of incubation at 37 °C. Kojic acid was used as a known tyrosinase inhibitor control. A450 of the samples containing tyrosinase solution, L-DOPA, and phosphate buffer only was set as 100% tyrosinase activity and used to calculate the percentage of enzyme activity in other samples. Each sample was analyzed in 3 independent repetitions.

### 3.9. Melanin Release Assay in B16F10

#### 3.9.1. Melanin Release from B16F10

B16 F10 cells were maintained in DMEM containing 4.5 g/L glucose, supplemented with 10% FBS. For the experiment, 5 × 104 B16F10 cells were seeded onto a well on a 6-well plate and grown for 3 days until the confluency of about 50%. At this point, the cells were treated with 1 µM α-MSH, 100 µg/mL kojic acid, or 100 µL/mL *A. biebersteinii* fractions in phenol red-free DMEM containing 10% FBS. Control cells were kept in culture medium without phenol red containing appropriate volumes of solvents. Following 3 days of culture, the culture medium was collected, centrifuged (5 min, 13,000 rpm), and the content of released melanin was measured spectrophotometrically at λ = 405 nm using FilterMax F5 microplate reader (Molecular Devices, San Jose, CA, USA).

#### 3.9.2. Cell Viability

Viability of B16F10 cells grown for 3 days, in the presence of 1 µM α-MSH 100 µg/mL kojic acid or *A. biebersteinii* fractions, was assessed by a Neutral Red Uptake Test [45]. Following the collection of culture medium containing released melanin, the cells were incubated for 3 h in medium containing 33 µg/mL neutral red. The cells were then washed with DPBS and lysed using acidified ethanol solution (50% ethanol, 1% acetic acid, 49% H_2_O). The absorbance of the released dye was measured at λ = 540 nm and corrected by the absorbance at λ = 620 nm. The value measured for control cells was set to 100% viability and used to calculate the percentage of viable cells in each experimental condition.

## 4. Conclusions

In this study, *Achillea biebersteinii* extracts and fractions were confirmed as a source of potential cosmetic ingredients with significant skin lightening properties and antioxidant activity. The influence of various extraction methods on the antioxidant and tyrosinase inhibitory properties was compared, indicating that ultrasonic and ASE extraction protocols might be used to produce *A. biebersteinii* extracts with the potential for cosmetic applications. This is also the first study showing the inhibitory activity of *A. biebersteinii* towards mammalian tyrosinase and melanin release from mammalian cells. Experimental data strongly indicate that caffeic acid derivatives are responsible for previously reported mushroom tyrosinase inhibitory activity of *A. bieberseinii* extracts [13]. However, with respect to mammalian tyrosinase, ferulic acid was shown to be the most promising inhibitory compound. Presented data, together with previously published findings on the antioxidant, antimicrobial, and wound healing properties of *A. biebersteinii* extracts strongly support the application of this species in cosmetic products [9,10,13]. The studies also proved that the selected experimental system plays a key role in the research on novel cosmetic ingredients with skin lightening potential.

## Figures and Tables

**Table 1:** The identified compounds in E6 extract from *Achillea biebersteinii* using an HPLC-ESI-Q-TOF-MS/MS (Rt = retention time, Delta = difference between experimental and calculated mass (mmu), DBE = double bond equivalents).

No.	Ionization Mode	Rt [min]	Molecular Formula	*m*/*z* Experimental	*m*/*z* Calculated	Delta [ppm]	DBE	Tentative Identification	MS/MS Fragments	Ref. **	Fraction No.
1	[M − H]^−^	6.9	C_5_H_8_O_4_	131.035	131.0350	−0.13	2	Glutaric acid	88,113		12
2	[M − H]^−^	7.0	C_15_H_18_O_3_	245.0465	245.1183	1.29	7	Achillin	211,179,171,101	[28]	
3	[M − H]^−^	7.1	C_6_H_12_O_7_	195.051	195.0510	0.13	1	Gluconic acid	59,71,129		12
4	[M − H]^−^	7.5	C_7_H_12_O_6_	191.0561	191.0561	0.006	2	Quinic acid	85,173,111	[26,29]	
5	[M − H]^−^	7.8	C_9_H_8_O_4_	179.0343	179.0350	3.79	6	Caffeic acid *	136	comparison with a standard, [13,27,30,31]	
6	[M − H]^−^	8.0	C_16_H_18_O_8_	337.0923	337.0929	10.33	8	Coumaroyl-quinic acid Isomers		[14]	
7	[M − H]^−^	8.3	C_4_H_5_O_5_	133.0138	133.0142	3.33	2	Malic acid	115, 89, 71		
8	[M − H]^−^	8.7	C_6_H_8_O_7_	191.0193	191.0197	2.22		Isocitric acid	173, 129,111,87		
9	[M − H]^−^	11.0	C_6_H_8_O_7_	191.0190	191.0197	3.78		Citric acid	129, 111, 87		
10	[M − H]^−^	12.4	C_4_H_6_O_4_	117.0193	117.0195	−1.42	2	Succinic acid	73,99,67		12
11	[M − H]^−^	14.5	C_7_H_10_O_5_	173.0455	173.0451	2.57	3	Shikimic acid	85,111,129		12
12	[M − H]^−^	14.6	C_16_H_18_O_10_	369.0819	369.0827	2.22	8	Fraxetin-8-*O*-glucoside	173,304,129,111		25,27
13	[M − H]^−^	15.0	C_13_H_16_O_9_	315.0704	315.0722	5.55	6	Protocatechuic acid glucoside	153,161,109		12
14	[M − H]^−^	15.3	C_16_H_18_O_9_	353.0867	353.0878	0.58	8	3-Caffeoylquinic acid *	191,173	comparison with a standard [13,14,31]	12
15	[M − H]^−^	15.2	C_15_H_18_O_9_	341.0886	341.0878	−2.32	7	Caffeoylglucoside (Isomer I)	251, 203, 179, 161	[32]	27
16	[M − H]^−^	15.7	C_15_H_18_O_9_	341.0899	341.0878	−6.12	7	Caffeoylglucoside (Isomer II)	281, 251, 179, 161	[32]	27
17	[M − H]^−^	15.8	C_26_H_28_O_14_	563.1401	563.1406	0.94	13	Schaftoside or isoschaftoside	353,191	[28,33]	25,27
18	[M − H]^−^	15.85	C_16_H_18_O_9_	353.0864	353.0878	0.02	8	5-Caffeoylquinic acid *	191,173	comparison with a standard [13,14,31]	12
19	[M − H]^−^	16.2	C_16_H_18_O_9_	353.0868	353.0878	2.84	8	4-Caffeoylquinic acid *	191,173	comparison with a standard [13,14,31]	12
20	[M − H]^−^	16.6	C_21_H_20_O_12_	477.0674	477.0675	0.13	13	Quercetin-*O*- glucopyranose	301,255,178,151		25
21	[M − H]^−^	16.9	C_25_H_24_O_12_	515.1178	515.1195	5.23	14	1,3-Dicaffeoylquinic acid (cynarin) *	353	comparison with a standard [13,14,31]	25,27
22	[M − H]^−^	17.9	C_17_H_26_O_4_	293.1768	293.1758	−3.29	5	Gmelinin B	111,193,163		25,27
23	[M − H]^−^	18.1	C_15_H_10_O_6_	285.0398	285.0405	2.31	11	Kaempferol	113,137	[14,26,27]	
24	[M − H]^−^	18.2	C_14_H_6_O_8_	300.9959	300.999	10.23	12	Ellagic acid	129, 179, 151,		
25	[M − H]^−^	18.3	C_10_H_10_O_4_	193.0536	193.0506	−15.29	6	Ferulic acid	161, 134	comparison with a standard [26,27,31]	25,27
26	[M − H]^−^	18.5	C_17_H_14_O_8_	345.0609	345.0616	4.6	11	Axillarin	315,287,129,81	[28]	
27	[M − H]^−^	19.7	C_18_H_16_O_8_	359.0772	359.0772	0.67	11	Jaceidin	301,258,286,344, 329	[28]	
28	[M + H]^+^	15.8	C_21_H_20_O_10_	433.2009	433.1129	2.58	12	Isovitexin	428,367	[28]	
29	[M + H]^+^	17.9	C_15_H_10_O_5_	271.0601	271.0601	0	11	Apigenin		[27,29,30,31]	

* indicates compounds previously identified in *A. biebersteinii* extract; ** compounds previously identified in *Achillea* species.

**Table 2:** Extraction conditions and DPPH scavenging activity of *A. biebersteinii* extracts (ASE—accelerated solvent extraction, UAE—ultrasound assisted extraction, M—maceration, SE—shaking extraction, RT—room temperature.

Code	Extraction Method	Extrahent	Temperature [ °C]	Time [min]
E1	ASE	75% EtOH	60	5
E2	ASE	75% EtOH	80	5
E3	ASE	75% EtOH	100	5
E4	ASE	75% EtOH	120	5
E5	ASE	75% EtOH	140	5
E6	ASE	75% EtOH	160	5
E7	ASE	75% EtOH	180	5
E8	ASE	75% EtOH	80	2
E9	ASE	75% EtOH	80	7
E10	ASE	75% EtOH	80	10
U1	UAE	75% EtOH	40	5
U2	UAE	75% EtOH	40	10
U3	UAE	75% EtOH	40	20
U4	UAE	75% EtOH	40	30
U5	UAE	75% EtOH	40	40
U6	UAE	75% EtOH	60	10
U7	UAE	75% EtOH	20	10
W1	SE	75% EtOH	RT	5
W2	SE	75% EtOH	RT	10
W3	SE	75% EtOH	RT	15
W4	SE	75% EtOH	RT	20
W5	SE	75% EtOH	RT	25
W6	SE	75% EtOH	RT	30
M1	MACERATION	75% EtOH	RT	300

## Data Availability

Data is contained within the article.

## Supplementary Materials

The supplementary materials are available online, Table S1: The MS/MS spectra of the identified components of *Achillea biebersteinii* extracts, Figure S1: The total ion chromatogram recorded for the E6 extract in the positive ionization mode.

## Sample Availability

Samples of compounds not available.
