# Supplementary material for: Identification of Mushroom and Murine Tyrosinase Inhibitors from Achillea biebersteinii Afan. Extract
Source: Molecules. 2021 Feb 11;26(4):964. doi: 10.3390/molecules26040964 (PMC7917799; doi:10.3390/molecules26040964)

molecules

MDPI

Article

Identification of Mushroom and Murine Tyrosinase Inhibitors from *Achillea biebersteinii* Afan. Extract

Marcelina Strzępek-Gomółka[1], Katarzyna Gaweł-Bęben[1,*], Apostolis Angelis[2], Beata Antosiewicz[1], Zuriyadda Sakipova[3], Kaldanay Kozhanova[3], Kazimierz Głowniak[1] and Wirginia Kukula-Koch[4]

[1] Department of Cosmetology, University of Information Technology and Management in Rzeszów, Sucharskiego 2, 35-225 Rzeszów, Poland, mstrzepek@wsiz.edu.pl (M. S.-G.), kagawel@wsiz.edu.pl (K. G.-B.), bantosiewicz@wsiz.edu.pl (B.A.), kglowniak@wsiz.edu.pl (K.G.)

[2] Laboratory of Pharmacognosy and Natural Products Chemistry, School of Pharmacy, National and Kapodistrian University of Athens, Panepistimioupoli Zografou, 15771 Athens, Greece; aangjel@pharm.uoa.gr (A.A.)

[3] School of Pharmacy, Kazakh National Medical University named after S.D. Asfendiyarov (KazNMU), , 88 Tole bi street, Almaty 050012, Kazakhstan, sakipova.z@kaznmu.kz, kozhanova.k@kaznmu.kz

[4] Chair and Department of Pharmacognosy, Medical University in Lublin, Chodźki 1, 20-093 Lublin, Poland, virginia.kukula@gmail.com

* Correspondence: kagawel@wsiz.edu.pl (K.G.-B.) Tel. 0048 17 866 1412

Table S1. The MS/MS spectra of the identified compolnents of *Achillea biebersteinii* extracts

<table>
<tr><td>Glutaric acid</td><td>
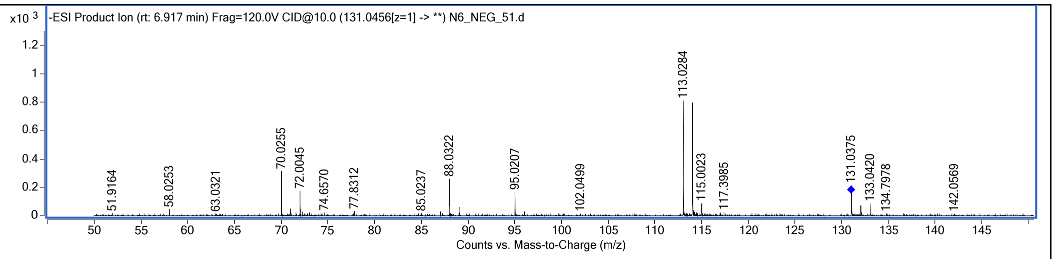

</td></tr>
<tr><td>Caffeic acid</td><td>
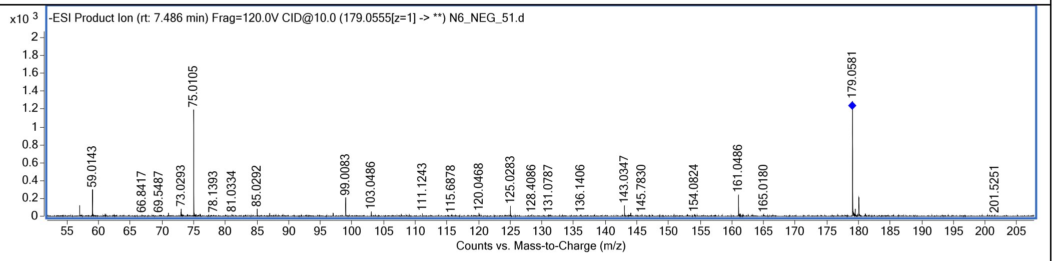

</td></tr>
<tr><td>Achillin</td><td>
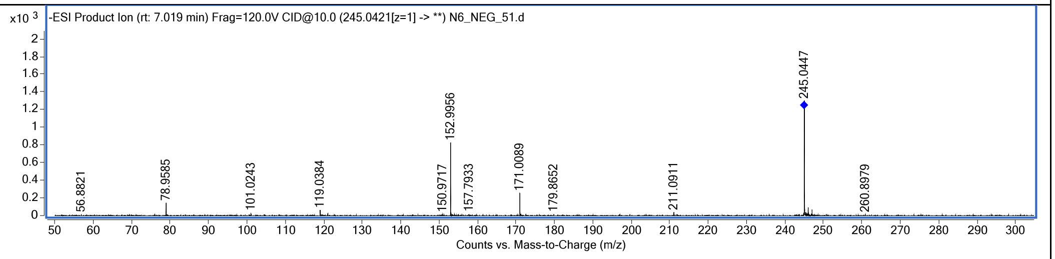

</td></tr>
</table>

Gluconic acid	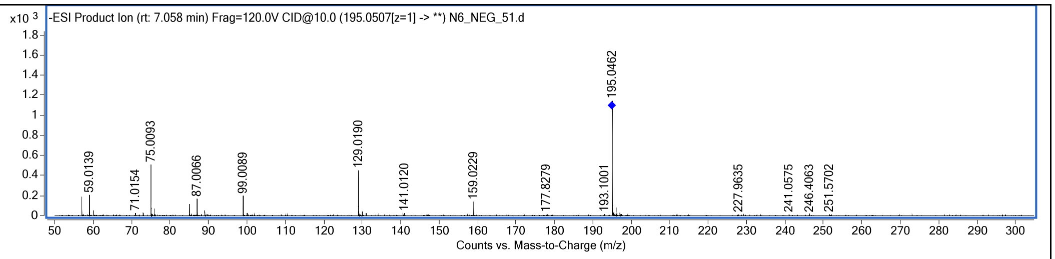
Quinic acid	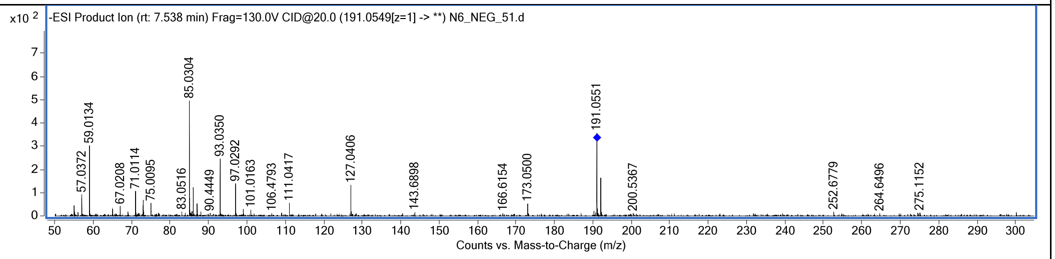
Succinic acid	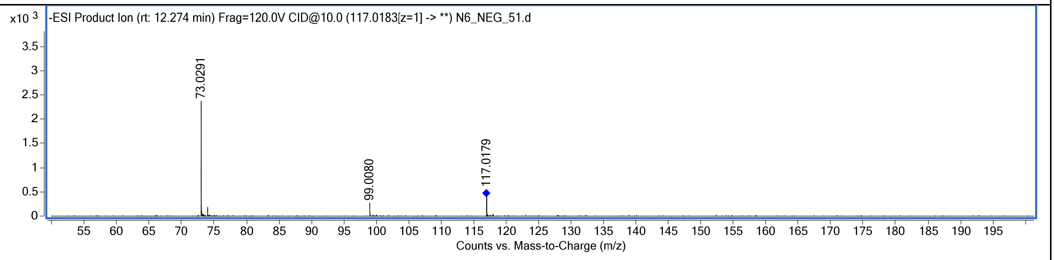
Shikimic acid	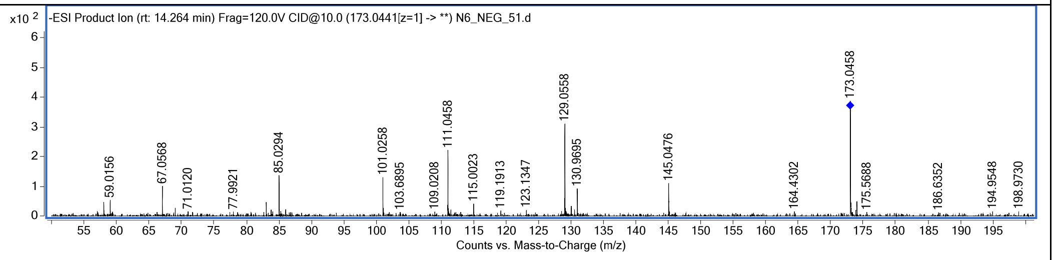
Fraxe-<br>tin-8-O-glucoside	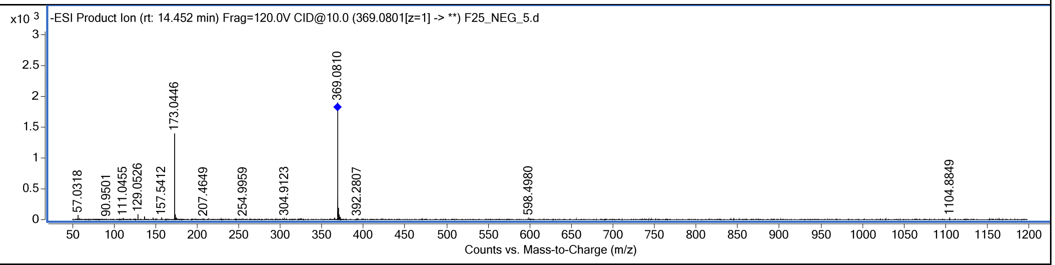

Protocatechuic acid glucoside	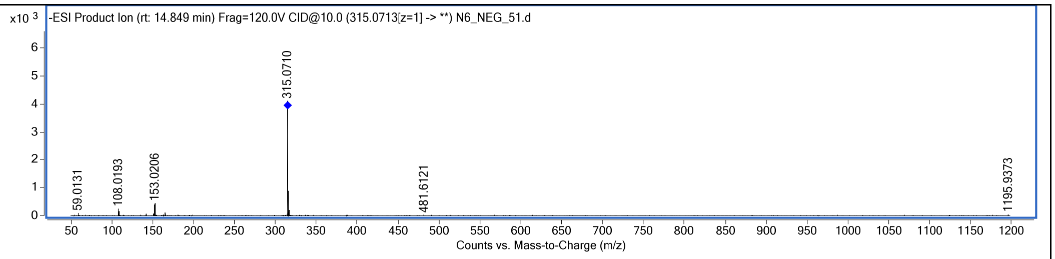
3-Caffeoylquinic acid	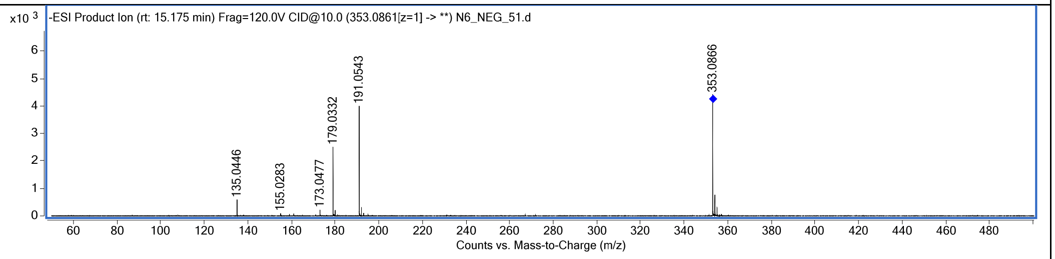
Caffeoylglucoside (Isomer I)	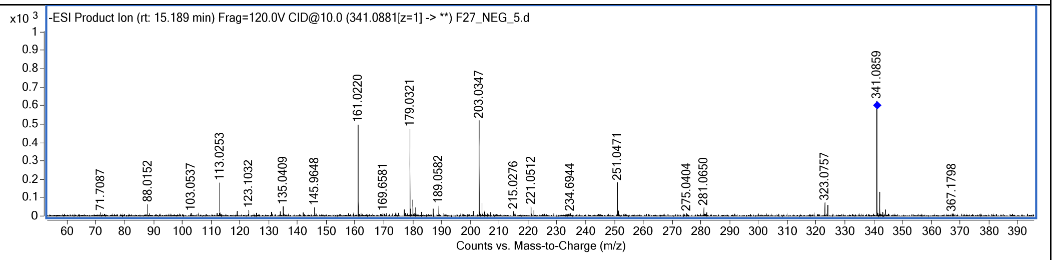
Caffeoylglucoside (Isomer II)	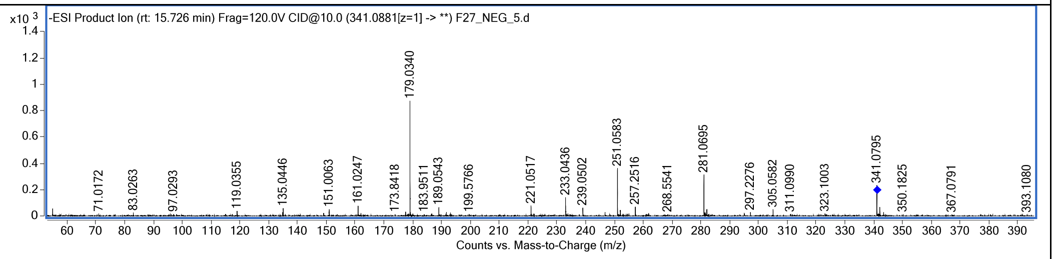
Schaftoside or isoschaftoside	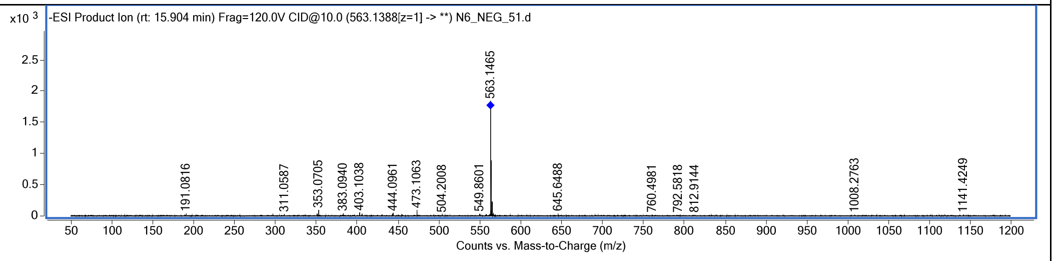

<table>
<tr><td>5-Caffeoylquinic acid</td><td>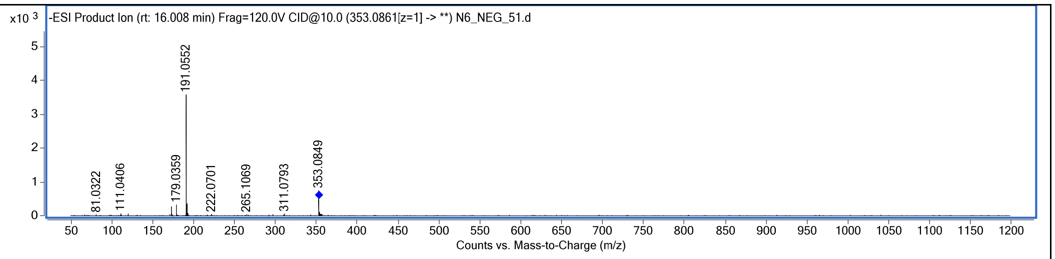
</td></tr>
<tr><td>4-Caffeoylquinic acid</td><td>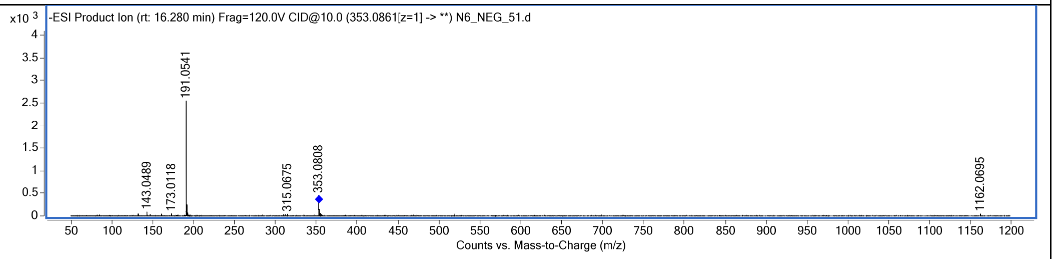
</td></tr>
<tr><td>Quercetin-O-glucopyranose</td><td>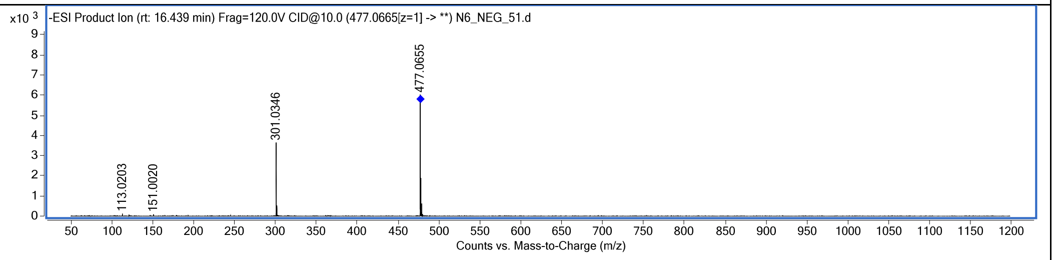
</td></tr>
<tr><td>1,3-Dicaffeoylquinic acid</td><td>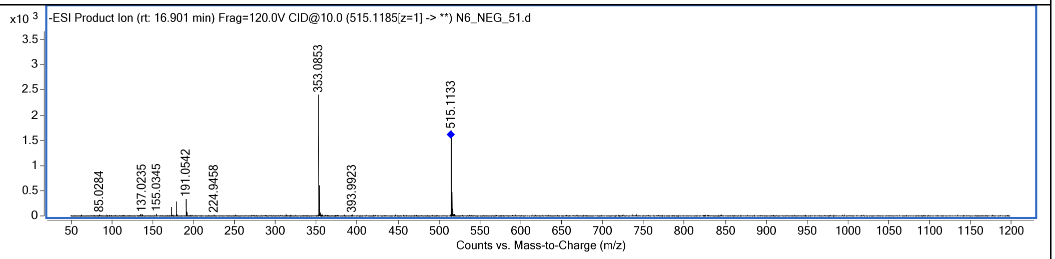
</td></tr>
<tr><td>Gmelinin B</td><td>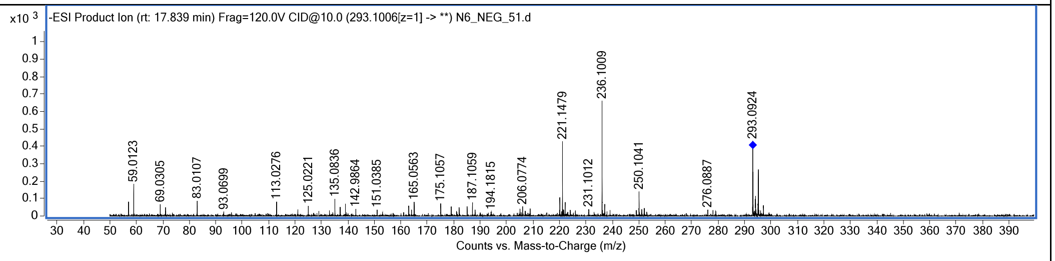
</td></tr>
</table>

Kaempferol	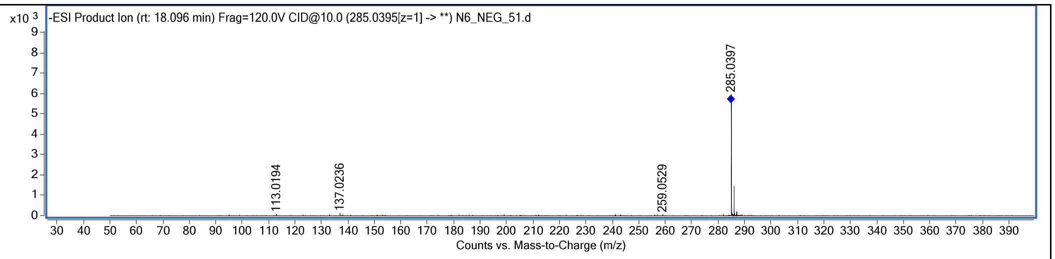
Ferulic acid	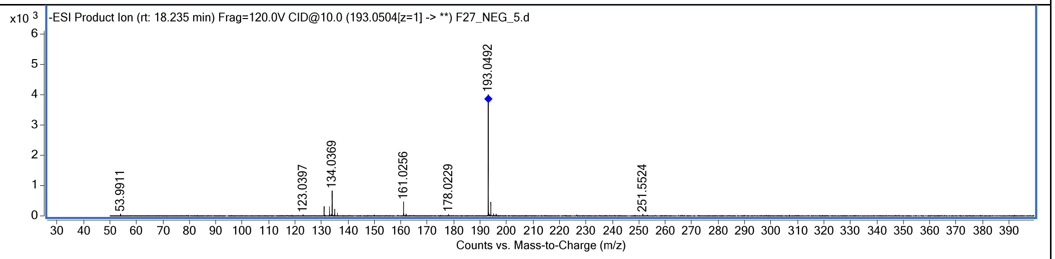
Ellagic acid	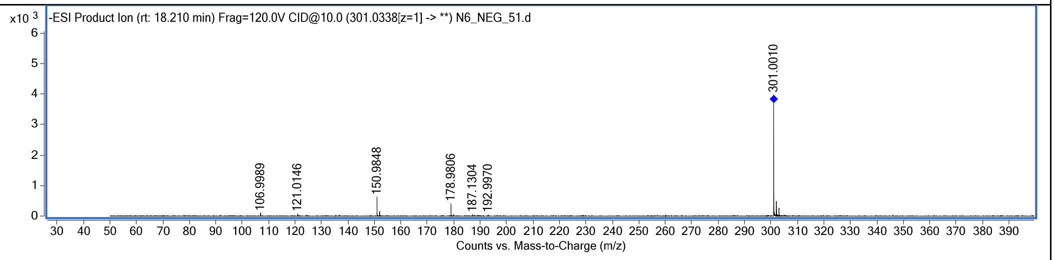
Axillarin	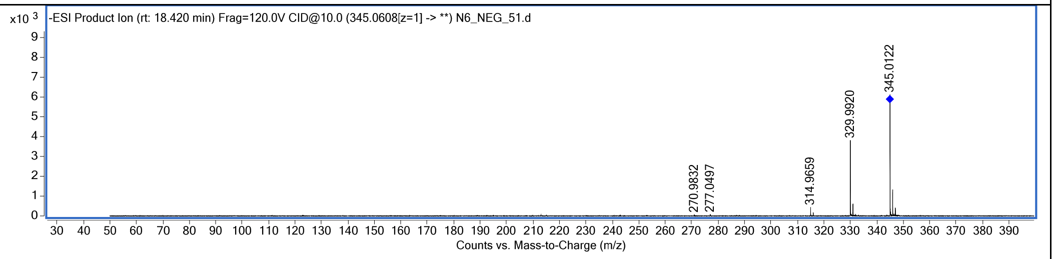
Jaceidin	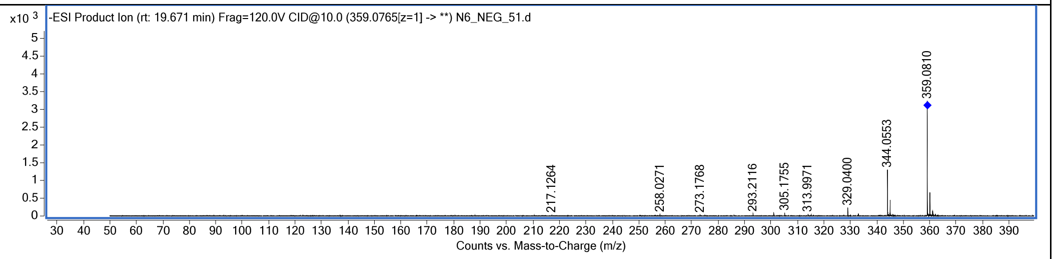

Isovitexin	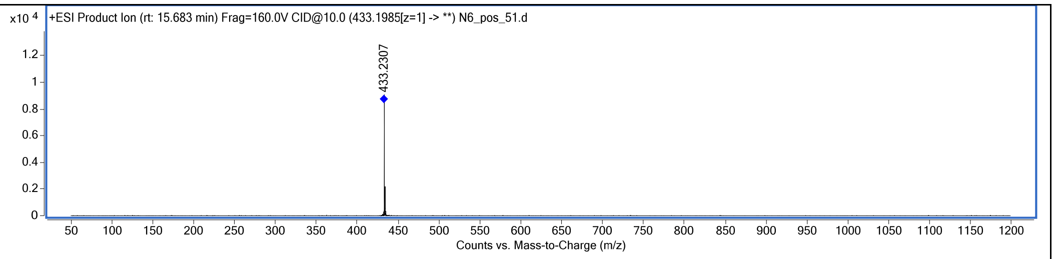
Malic acid	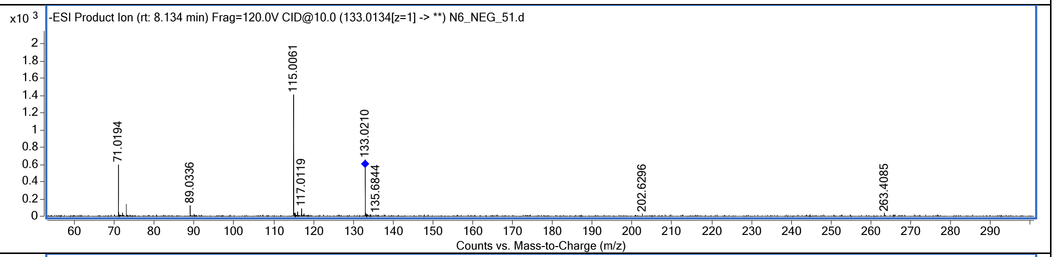
Isocitric acid	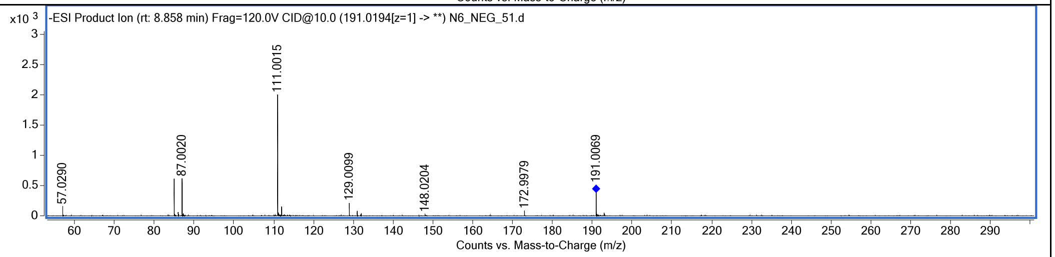
Citric acid	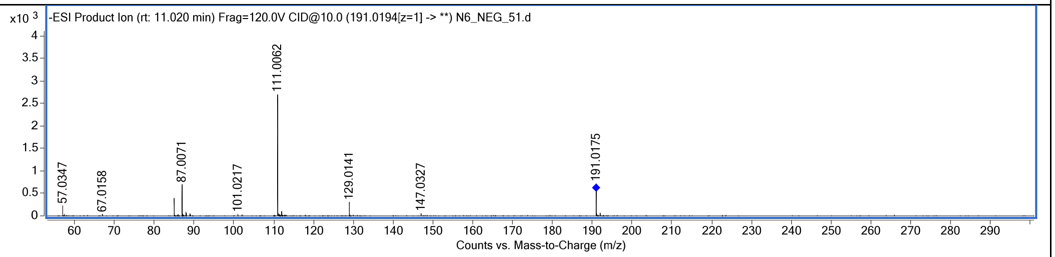

Figure S1. The total ion chromatogram recorded for the E6 extract in the positive ionization mode.

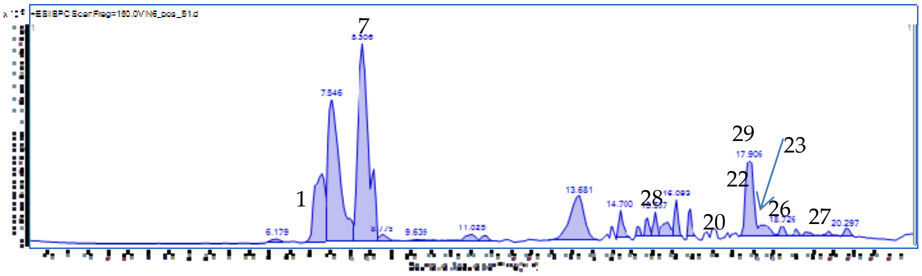

Supplement: Supplementary file 1 [file molecules-26-00964-s001.pdf]
